# Supplementary material for: Semliki Forest Virus (SFV) Self-Amplifying RNA Delivered to J774A.1 Macrophage Lineage by Its Association with a Purified Recombinant SFV Capsid Protein
Source: Int J Mol Sci. 2024 Jul 18;25(14):7859. doi: 10.3390/ijms25147859 (PMC11276834; doi:10.3390/ijms25147859)
Supplement: Supplementary file 1 [file ijms-25-07859-s001.zip › ijms-3001526-supplementary.pdf]

Expression of C protein in *E. coli* Rosetta strains

The plasmid containing the C protein gene under the control of T7 promoter was inserted into *E. coli* Rosetta (DE3) and *E. coli* Rosetta pLyss in order to check if the expression of the low molecular weight product could be decreased or eliminated. Results clearly show that the expression in both bacteria strains did not change the expression profile in relation to the previously used BL21 DE3 strain (Figure S1).

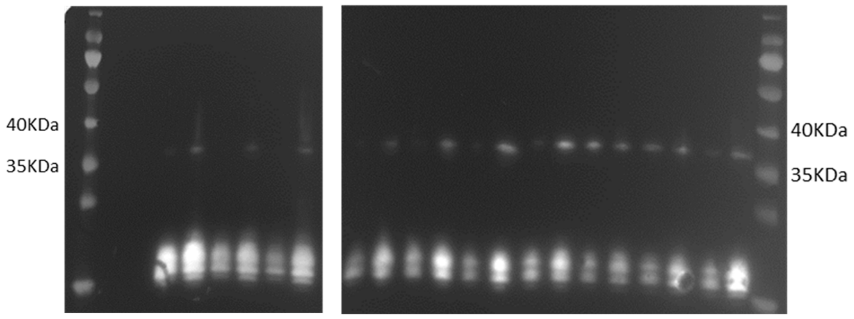

Figure S1. Expression of C protein in *E. coli* Rosetta (DE3) and *E. coli* Rosetta pLyss at 28 °C. Western blotting analysis show the expression of the C protein at the size of 35 kDa as well as the truncated form of around 20 kDa in every clone tested in *E. coli* Rosetta (DE3) (left) and *E. coli* Rosetta pLyss (right). Samples of cell pellet were analyzed by SDS-PAGE 12% followed by Western blotting using anti-6x histidine tag HRP-conjugated antibody labeling. Membranes were revealed with West Pico ECL kit (Pierce) and documented.

Influence of IPTG concentration on the expression of C protein and its truncated form

The clone used for experimentation (clone 2, Figure 1) was cultivated at 37 °C until it reached the appropriate DO when it was induced with one of two different IPTG concentrations (0.01 mM or 0.5 mM) and incubated at 28 °C. Samples taken from 1 hour to 5 hours after induction showed no difference in the pattern of C protein expression regarding concentration and presence of the truncated form (Figure S2).

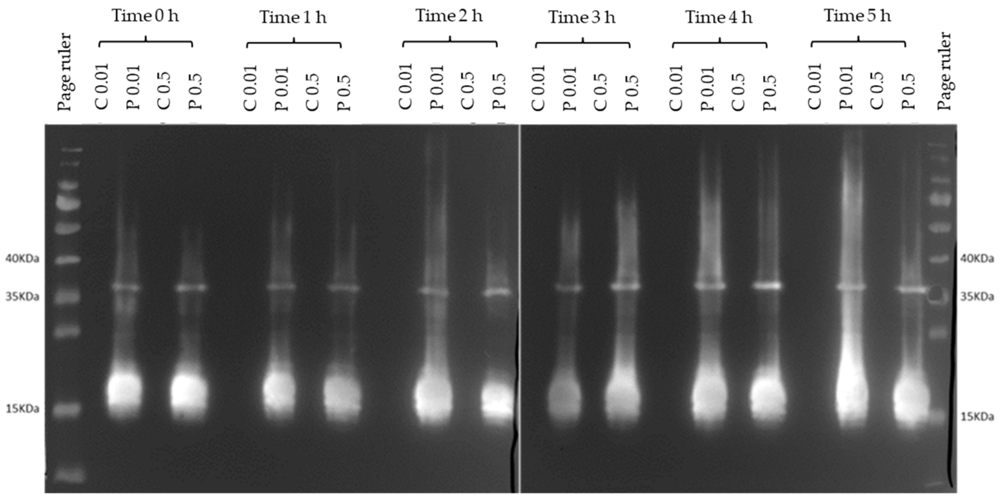

Figure S2. Expression of C protein after induction with different IPTG concentrations. *E. coli* BL21 were induced to express C protein at 28 °C under the presence of IPTG at 0.01 or 0.5 mM. Samples were taken after one hour of induction and analyzed by SDS-PAGE 12% followed by Western

blotting using anti-6x histidine tag HRP-conjugated antibody labeling. Membranes were revealed with West Pico ECL kit (Pierce) and documented.
